# Chicago Medical, Meeting August 3d

**Published:** 1885-09

**Authors:** 


### SOCIETY PROCEEDINGS.

CHICAGO MEDICAL SOCIETY.

*Stated Meeting, August 3rd, 1885.* — THE PRESIDENT, PROFESSOR CHARLES T. PARKES, M. D., in the chair.

DR. FRANKLIN H. MARTIN read a paper on the "Oleate of *Manganese.*" He said: There is little doubt left in the minds of therapeutists in regard to the value of manganese as a remedy in certain forms of menstrual trouble. The remedy, in the form of permanganate of potash, was first brought to the attention of the profession by Ringer and Murrell, of London, in the spring of 1883. They recommended the drug in functional amenorrhœa. Soon after this he commenced experiments with the same preparations, and published the results in *The New York Medical Record,* Sept. 29, 1883. To his knowledge, that was the first that anything on the subject was published in this country.

In the course of his experiments, acting on the theory that the drug produced menstruation by stimulating the menstrual organs, he was induced to give the remedy in menorrhagia and metrorrhagia dependent upon an *atonic* condition of the organ. He found to his gratification that it acted equally well in these conditions, as in the opposite. He has also obtained good results from its administration in irregularities incident to approaching menopause. He has received very gratifying letters from many members of the profession throughout the country, who have used the drug in one or more of the conditions mentioned above, with good results.

Professor ETHERIDGE thought much advance would be made in the use of drugs if we were more careful to discover in what conditions they were beneficial. He had found this remedy useful in cases of atonic amenorrhœa, with the uterus in its normal position. He had found the aqueous solution an eligible preparation, and had also used it in the form of a suppository.

Dr. E. J. DOERING, said he had used this remedy in some cases of atonic amenorrhœa with good results. He asked the effect of the drug upon the pregnant uterus.

PROFESSOR PAOLI had used manganese somewhat for several years, in cases of menstrual disorder, with varying degrees of success. It was a useful remedy in many skin diseases. He had not used the oleate, and could not see how it acted upon the uterus, unless, possibly, by being applied at once after being freshly prepared.

DR. MARTIN closed the discussion by saying he had used the aqueous solution in very small doses, also had had the remedy put in dry papers and swallowed with a glassful of water. He had never used it in the form of a suppository. Manganese has no effect on the pregnant uterus. The drug seems to act as a general stimulant to the uterus, causing it to perform its normal function. It might be absorbed as the oleate and so produce its effects.

"*Does the Use of Tobacco Injure Sight?*" was the subject of a paper read by W. FRANKLIN COLEMAN, M. D.

To consider the physical effects of a poisonous weed, food for neither man nor beast, yet consumed alike in every quarter of the globe, by savage and sage, saint and sinner, is a theme of no little consequence. The active principles of tobacco are an alkaloid called nicotine and a volatile oil called nicotianin; nicotine is also contained in tobacco-smoke. The observations of Claude Bernard that "nicotine at first produces contraction of the arteries, and later on the vessels become distended," agree with the views of Uspensky, who from physiological researches concludes that "Nicotine first stimulates then paralyzes the vaso-motor centers."

From personal experience, and the literature at his command, he knows of no more constant detrimental effect of the use of tobacco than more or less impairment of vision. In England there is a general belief amongst surgeons that tobacco is more frequently the cause of amblyopia, while in America it is attributed more generally to alcohol.

In regard to treatment, the withholding of tobacco is an evident requisite. Opinion is divided as to the benefit of the hypodermic use of strychnia. He believes that if the strychnia is pushed to the production of spasms, usually requiring 1-10 gr., often 1/8 and occasionally 1/4 gr., twice a day, recovery is always hastened and often occasioned.

The great majority of the authors who have written on diseases of the eye agree that amblyopia is often caused by the use of tobacco. That the Turks, who use a large amount of this weed, are not oftener subject to disorders of vision, may be due to their habit of using long stems to their pipes, or to the use of the nargileh or water-pipe, by which means the amount of nicotine that reaches the mouth is much reduced.

Peculiarity of race, idiosyncracy of constitution, nervous exhaustion, impaired nutrition and other debilitating causes may or may not account for the elective affinity of tobacco for the eyes of certain individuals; but to deny that tobacco produces amblyopia because so large a number smoke their poison with impunity, is as reasonable as to deny that cirrhosis of the liver is caused by alcohol, because so many dram-drinkers maintain sound livers; or that cold and wet can produce rheumatism, because so few of the exposed multitude suffer.

DR. PAOLI said while the excessive indulgence in tobacco might be harmful, the fact of its almost universal use and continued use in many cases for years resulted in no deleterious effects, would seem to show that its narcotic power, used in the ordinary way, was very slight. He had used tobacco for fifty-five years and felt no ill results. The Germans use large quantities of it, and do not suffer. This seemed to be a case of excess of zeal on the part of the ophthalmologists to discover a cause for amaurosis. We ought to be conservative on this point.

DR. COLEMAN closed by saying that only in certain susceptible conditions was tobacco liable to cause disorders of vision. But that so few suffered was no proof that it had not this baneful influence when acting with other causes.

### LACERATION OF THE CERVIX UTERI.

DR. JOHN BARTLETT read a paper in which he said his object in addressing the Society was to suggest a way and a time in which laceration of the cervix uteri may be easily and certainly detected soon after its occurrence.

Directly after delivery, if the fingers be introduced deeply into the vagina up to the contracted os uteri internum, and then carried in any direction a little outwardly, the flabby and floating ring formed by the non-contracted cervix may be felt, as Guillemeau described it three hundred years ago, "like a section of large intestine."

By very carefully following the entire circumference of this ring an existing rent may be discovered. But this examination is attended with some difficulties. The patient is exhausted with her labour, and fatigued with attentions, and just now, since "it is all over," longing for rest. She is impatient of, and perturbed, by this *post factum* inquiry. Her state of mind, and possible expression of complaint, are apt to render an examination, which the physician cannot regard as absolutely necessary, less exact and thorough than it would be otherwise. And then, the soft and floating margins of the cervix have often somewhat of an intangible feel, if the expression be permitted, gliding past the fingers like a detached clot of blood, and occasionally, in some portion of their circumference, passing out of satisfactory reach.

On this account it is not surprising to hear an obstetrician say that he cannot tell whether the post-partum cervix is lacerated or not. The error of the accoucheurs who fail to recognize such a condition is, that they do not make their observation of the suspected cervix at the proper time. They examine the neck actually, as has just been done mentally—after the clearance of the uterus. The favorable moment for the examination—and that he said was the special point of his remarks—is just as the placenta is beginning to occupy and distend the cervix. The collar of flesh is then not floating and uncertain in feel, but stretched and expanded, forming a distinct ring, easily followed in its entire circumference. At this moment then, just as the cervical tube is being rendered tense by the placental mass, any laceration in it may be detected with ease and certainty.

PROFESSOR ETHERIDGE asked the author of the paper whether he had verified a case by speculum examination after discovering it in the way he had illustrated in his remarks.

PROFESSOR PARKES said he had great difficulty in detecting laceration after delivery, on account of the relaxed condition of the parts. He thought the suggestion of DR. BARTLETT as to the way to obviate this difficulty was a good one.

DR. DOERING inquired as to the size of lacerations he had found.

DR. BARTLETT concluded by saying that he had verified cases of laceration discovered in the manner proposed by him. The largest laceration he had found was one and-a-half inches in length, and the end of the little finger could be passed into it. In one case he encountered considerable hemorrhage from such a rent: and this may be the cause of continuous loss of blood when the os is well contracted.

DR. BARTLETT illustrated his remarks with earthen-ware models, turned by a potter, under his immediate direction.

The society then adjourned.

